# Chapter 11: Genome-Wide Association Studies

**DOI:** 10.1371/journal.pcbi.1002822

**Published:** 2012-12-27

**Authors:** William S. Bush, Jason H. Moore

**Affiliations:** 1 Department of Biomedical Informatics, Center for Human Genetics Research, Vanderbilt University Medical School, Nashville, Tennessee, United States of America; 2 Departments of Genetics and Community Family Medicine, Institute for Quantitative Biomedical Sciences, Dartmouth Medical School, Lebanon, New Hampshire, United States of America; Whitehead Institute, United States of America; University of Maryland, Baltimore County, United States of America

## Abstract

Genome-wide association studies (GWAS) have evolved over the last ten years into a powerful tool for investigating the genetic architecture of human disease. In this work, we review the key concepts underlying GWAS, including the architecture of common diseases, the structure of common human genetic variation, technologies for capturing genetic information, study designs, and the statistical methods used for data analysis. We also look forward to the future beyond GWAS.

**What to Learn in This Chapter**

- Basic genetic concepts that drive genome-wide association studies
- Genotyping technologies and common study designs
- Statistical concepts for GWAS analysis
- Replication, interpretation, and follow-up of association results

This article is part of the “Translational Bioinformatics” collection for *PLOS Computational Biology*.

## 1. Important Questions in Human Genetics

A central goal of human genetics is to identify genetic risk factors for common, complex diseases such as schizophrenia and type II diabetes, and for rare Mendelian diseases such as cystic fibrosis and sickle cell anemia. There are many different technologies, study designs and analytical tools for identifying genetic risk factors. We will focus here on the genome-wide association study or GWAS that measures and analyzes DNA sequence variations from across the human genome in an effort to identify genetic risk factors for diseases that are common in the population. The ultimate goal of GWAS is to use genetic risk factors to make predictions about who is at risk and to identify the biological underpinnings of disease susceptibility for developing new prevention and treatment strategies. One of the early successes of GWAS was the identification of the *Complement Factor H* gene as a major risk factor for age-related macular degeneration or AMD [1]–[3]. Not only were DNA sequence variations in this gene associated with AMD but the biological basis for the effect was demonstrated. Understanding the biological basis of genetic effects will play an important role in developing new pharmacologic therapies.

While understanding the complexity of human health and disease is an important objective, it is not the only focus of human genetics. Accordingly, one of the most successful applications of GWAS has been in the area of pharmacology. Pharmacogenetics has the goal of identifying DNA sequence variations that are associated with drug metabolism and efficacy as well as adverse effects. For example, warfarin is a blood-thinning drug that helps prevent blood clots in patients. Determining the appropriate dose for each patient is important and believed to be partly controlled by genes. A recent GWAS revealed DNA sequence variations in several genes that have a large influence on warfarin dosing [4]. These results, and more recent validation studies, have led to genetic tests for warfarin dosing that can be used in a clinical setting. This type of genetic test has given rise to a new field called *personalized medicine* that aims to tailor healthcare to individual patients based on their genetic background and other biological features. The widespread availability of low-cost technology for measuring an individual's genetic background has been harnessed by businesses that are now marketing genetic testing directly to the consumer. Genome-wide association studies, for better or for worse, have ushered in the exciting era of personalized medicine and personal genetic testing. The goal of this chapter is to introduce and review GWAS technology, study design and analytical strategies as an important example of translational bioinformatics. We focus here on the application of GWAS to common diseases that have a complex multifactorial etiology.

## 2. Concepts Underlying the Study Design

### 2.1 Single Nucleotide Polymorphisms

The modern unit of genetic variation is the *single nucleotide polymorphism* or SNP. SNPs are single base-pair changes in the DNA sequence that occur with high frequency in the human genome [5]. For the purposes of genetic studies, SNPs are typically used as *markers* of a genomic region, with the large majority of them having a minimal impact on biological systems. SNPs can have functional consequences, however, causing amino acid changes, changes to mRNA transcript stability, and changes to transcription factor binding affinity [6]. SNPs are by far the most abundant form of genetic variation in the human genome.

SNPs are notably a type of *common* genetic variation; many SNPs are present in a large proportion of human populations [7]. SNPs typically have two alleles, meaning within a population there are two commonly occurring base-pair possibilities for a SNP location. The frequency of a SNP is given in terms of the *minor allele frequency* or the frequency of the less common allele. For example, a SNP with a minor allele (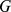) frequency of 0.40 implies that 40% of a population has the 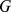 allele versus the more common allele (the major allele), which is found in 60% of the population.

Commonly occurring SNPs lie in stark contrast to genetic variants that are implicated in more rare genetic disorders, such as cystic fibrosis [8]. These conditions are largely caused by extremely rare genetic variants that ultimately induce a detrimental change to protein function, which leads to the disease state. Variants with such low frequency in the population are sometimes referred to as *mutations*, though they can be structurally equivalent to SNPs - single base-pair changes in the DNA sequence. In the genetics literature, the term SNP is generally applied to *common* single base-pair changes, and the term mutation is applied to *rare* genetic variants.

### 2.2 Failures of Linkage for Complex Disease

Cystic fibrosis (and most rare genetic disorders) can be caused by multiple different genetic variants within a single gene. Because the effect of the genetic variants is so strong, cystic fibrosis follows an autosomal dominant inheritance pattern in families with the disorder. One of the major successes of human genetics was the identification of multiple mutations in the *CFTR* gene as the cause of cystic fibrosis [8]. This was achieved by genotyping families affected by cystic fibrosis using a collection of genetic markers across the genome, and examining how those genetic markers segregate with the disease across multiple families. This technique, called *linkage analysis*, was subsequently applied successfully to identify genetic variants that contribute to rare disorders like Huntington disease [9]. When applied to more common disorders, like heart disease or various forms of cancer, linkage analysis has not fared as well. This implies the genetic mechanisms that influence common disorders are different from those that cause rare disorders [10].

### 2.3 Common Disease Common Variant Hypothesis

The idea that common diseases have a different underlying genetic architecture than rare disorders, coupled with the discovery of several susceptibility variants for common disease with high minor allele frequency (including alleles in the *apolipoprotein E* or *APOE* gene for Alzheimer's disease [11] and *PPARg* gene in type II diabetes [12]), led to the development of the *common disease/common variant* (CD/CV) hypothesis [13].

This hypothesis states simply that common disorders are likely influenced by genetic variation that is also common in the population. There are several key ramifications of this for the study of complex disease. First, if common genetic variants influence disease, the effect size (or penetrance) for any one variant must be small relative to that found for rare disorders. For example, if a SNP with 40% frequency in the population causes a highly deleterious amino acid substitution that directly leads to a disease phenotype, nearly 40% of the population would have that phenotype. Thus, the allele frequency and the population prevalence are completely correlated. If, however, that same SNP caused a small change in gene expression that alters risk for a disease by some small amount, the prevalence of the disease and the influential allele would be only slightly correlated. As such, common variants almost by definition cannot have high penetrance.

Secondly, if common alleles have small genetic effects (low penetrance), but common disorders show heritability (inheritance in families), then multiple common alleles must influence disease susceptibility. For example, twin studies might estimate the heritability of a common disease to be 40%, that is, 40% of the total variance in disease risk is due to genetic factors. If the allele of a single SNP incurs only a small degree of disease risk, that SNP only explains a small proportion of the total variance due to genetic factors. As such, the total genetic risk due to common genetic variation must be spread across multiple genetic factors. These two points suggest that traditional family-based genetic studies are not likely to be successful for complex diseases, prompting a shift toward population-based studies.

**Figure 1:**
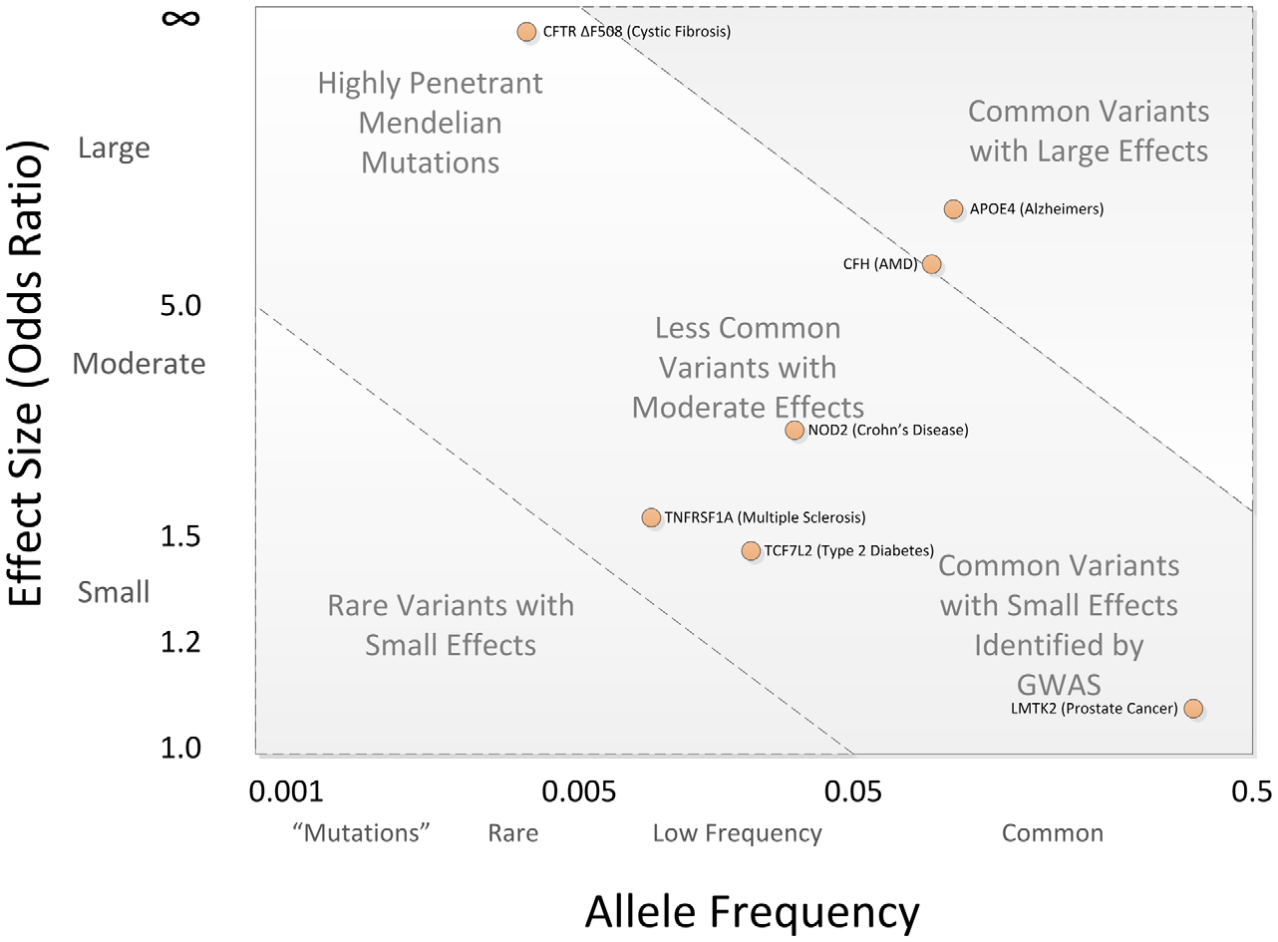
Spectrum of Disease Allele Effects. Disease associations are often conceptualized in two dimensions: allele frequency and effect size. Highly penetrant alleles for Mendelian disorders are extremely rare with large effect sizes (upper left), while most GWAS findings are associations of common SNPs with small effect sizes (lower right). The bulk of discovered genetic associations lie on the diagonal denoted by the dashed lines.

The frequency with which an allele occurs in the population and the risk incurred by that allele for complex diseases are key components to consider when planning a genetic study, impacting the technology needed to gather genetic information and the sample size needed to discover statistically significant genetic effects. The spectrum of potential genetic effects is sometimes visualized and partitioned by effect size and allele frequency (figure 1). Genetic effects in the upper right are more amenable to smaller family-based studies and linkage analysis, and may require genotyping relatively few genetic markers. Effects in the lower right are typical of findings from GWAS, requiring large sample sizes and a large panel of genetic markers. Effects in the upper right, most notably *CFH*, have been identified using both linkage analysis and GWAS. Effects in the lower left are perhaps the most difficult challenge, requiring genomic sequencing of large samples to associate rare variants to disease.

Over the last five years, the common disease/common variant hypothesis has been tested for a variety of common diseases, and while much of the heritability for these conditions is not yet explained, common alleles certainly play a role in susceptibility. The National Human Genome Institute GWAS catalog (http://www.genome.gov/gwastudies) lists over 3,600 SNPs identified for common diseases or traits, and in general, common diseases have multiple susceptibility alleles, each with small effect sizes (typically increasing disease risk between 1.2–2 times the population risk) [14]. From these results we can say that for most common diseases, the CD/CV hypothesis is true, though it should not be assumed that the *entire* genetic component of any common disease is due to common alleles only.

## 3. Capturing Common Variation

### 3.1 The Human Haplotype Map Project

To test the common disease/common variant hypothesis for a phenotype, a systematic approach is needed to interrogate much of the common variation in the human genome. First, the location and density of commonly occurring SNPs is needed to identify the genomic regions and individual sites that must be examined by genetic studies. Secondly, population-specific differences in genetic variation must be cataloged so that studies of phenotypes in different populations can be conducted with the proper design. Finally, correlations among common genetic variants must be determined so that genetic studies do not collect redundant information. The International HapMap Project was designed to identify variation across the genome and to characterize correlations among variants.

The International HapMap Project used a variety of sequencing techniques to discover and catalog SNPs in European descent populations, the Yoruba population of African origin, Han Chinese individuals from Beijing, and Japanese individuals from Tokyo [15], [16]. The project has since been expanded to include 11 human populations, with genotypes for 1.6 million SNPs [7]. HapMap genotype data allowed the examination of *linkage disequilibrium*.

### 3.2 Linkage Disequilibrium

**Figure 2:**
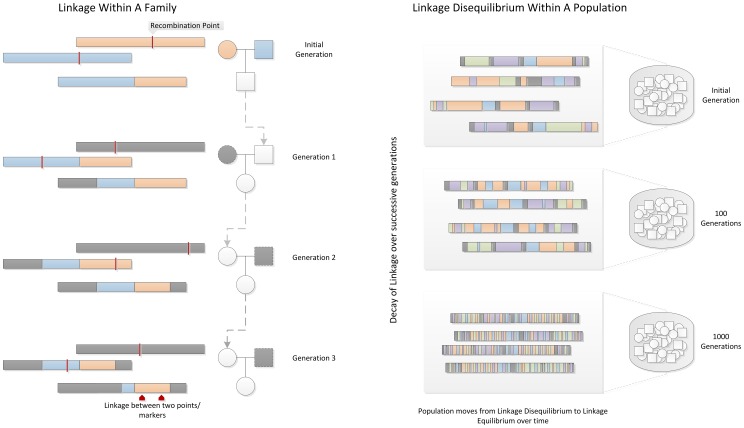
Linkage and Linkage Disequilibrium. Within a family, linkage occurs when two genetic markers (points on a chromosome) remain linked on a chromosome rather than being broken apart by recombination events during meiosis, shown as red lines. In a population, contiguous stretches of founder chromosomes from the initial generation are sequentially reduced in size by recombination events. Over time, a pair of markers or points on a chromosome in the population move from linkage disequilibrium to linkage equilibrium, as recombination events eventually occur between every possible point on the chromosome.

Linkage disequilibrium (LD) is a property of SNPs on a contiguous stretch of genomic sequence that describes the degree to which an allele of one SNP is inherited or correlated with an allele of another SNP within a population. The term linkage disequilibrium was coined by population geneticists in an attempt to mathematically describe changes in genetic variation within a population over time. It is related to the concept of *chromosomal linkage*, where two markers on a chromosome remain physically joined on a chromosome through generations of a family. In figure 2, two founder chromosomes are shown (one in blue and one in orange). Recombination events within a family from generation to generation break apart chromosomal segments. This effect is amplified through generations, and in a population of fixed size undergoing random mating, repeated random recombination events will break apart segments of contiguous chromosome (containing linked alleles) until eventually all alleles in the population are in *linkage equilibrium* or are independent. Thus, linkage between markers on a population scale is referred to as *linkage disequilibrium*.

The rate of LD decay is dependent on multiple factors, including the population size, the number of founding chromosomes in the population, and the number of generations for which the population has existed. As such, different human sub-populations have different degrees and patterns of LD. African-descent populations are the most ancestral and have smaller regions of LD due to the accumulation of more recombination events in that group. European-descent and Asian-descent populations were created by founder events (a sampling of chromosomes from the African population), which altered the number of founding chromosomes, the population size, and the generational age of the population. These populations on average have larger regions of LD than African-descent groups.

Many measures of LD have been proposed [17], though all are ultimately related to the difference between the observed frequency of co-occurrence for two alleles (i.e. a two-marker haplotype) and the frequency expected if the two markers are independent. The two commonly used measures of linkage disequilibrium are 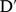 and 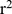 [15], [17] shown in equations 1 and 2. In these equations, 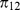 is the frequency of the 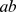 haplotype, 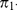 is the frequency of the 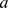 allele, and 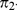 is the frequency of the 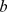 allele.
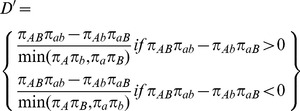
(1)

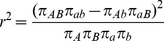
(2)

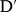
 is a population genetics measure that is related to recombination events between markers and is scaled between 0 and 1. A 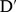 value of 0 indicates complete linkage equilibrium, which implies frequent recombination between the two markers and statistical independence under principles of Hardy-Weinberg equilibrium. A 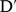 of 1 indicates complete LD, indicating no recombination between the two markers within the population. For the purposes of genetic analysis, LD is generally reported in terms of 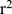, a statistical measure of correlation. High 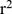 values indicate that two SNPs convey similar information, as one allele of the first SNP is often observed with one allele of the second SNP, so only one of the two SNPs needs to be genotyped to capture the allelic variation. There are dependencies between these two statistics; 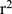 is sensitive to the allele frequencies of the tow markers, and can only be high in regions of high 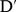.

One often forgotten issue associated with LD measures is that current technology does not allow direct measurement of haplotype frequencies from a sample because each SNP is genotyped independently and the *phase* or chromosome of origin for each allele is unknown. Many well-developed and documented methods for inferring haplotype phase and estimating the subsequent two-marker haplotype frequencies exist, and generally lead to reasonable results [18].

SNPs that are selected specifically to capture the variation at nearby sites in the genome are called *tag SNPs* because alleles for these SNPs tag the surrounding stretch of LD. As noted before, patterns of LD are population specific and as such, tag SNPs selected for one population may not work well for a different population. LD is exploited to optimize genetic studies, preventing genotyping SNPs that provide redundant information. Based on analysis of data from the HapMap project, >80% of commonly occurring SNPs in European descent populations can be captured using a subset of 500,000 to one million SNPs scattered across the genome [19].

### 3.3 Indirect Association

**Figure 3:**
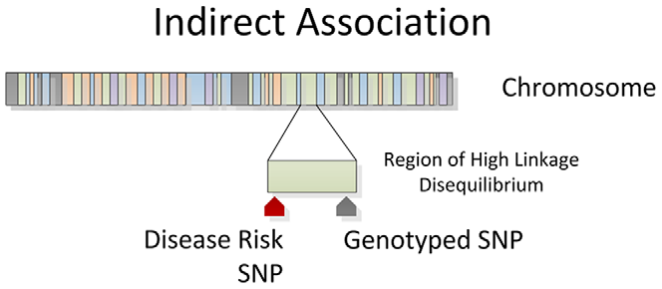
Indirect Association. Genotyped SNPs often lie in a region of high linkage disequilibrium with an influential allele. The genotyped SNP will be statistically associated with disease as a surrogate for the disease SNP through an indirect association.

The presence of LD creates two possible positive outcomes from a genetic association study. In the first outcome, the SNP influencing a biological system that ultimately leads to the phenotype is directly genotyped in the study and found to be statistically associated with the trait. This is referred to as a *direct association*, and the genotyped SNP is sometimes referred to as the *functional SNP*. The second possibility is that the influential SNP is not directly typed, but instead a tag SNP in high LD with the influential SNP is typed and statistically associated to the phenotype (figure 3). This is referred to as an *indirect association* [10]. Because of these two possibilities, a significant SNP association from a GWAS should not be assumed as the causal variant and may require additional studies to map the precise location of the influential SNP.

Conceptually, the end result of GWAS under the common disease/common variant hypothesis is that a panel of 500,000 to one million markers will identify common SNPs that are associated to common phenotypes. To conduct such a study practically requires a genotyping technology that can accurately capture the alleles of 500,000 to one million SNPs for each individual in a study in a cost-effective manner.

## 4. Genotyping Technologies

Genome-wide association studies were made possible by the availability of chip-based microarray technology for assaying one million or more SNPs. Two primary platforms have been used for most GWAS. These include products from Illumina (San Diego, CA) and Affymetrix (Santa Clara, CA). These two competing technologies have been recently reviewed [20] and offer different approaches to measure SNP variation. For example, the Affymetrix platform prints short DNA sequences as a spot on the chip that recognizes a specific SNP allele. Alleles (i.e. nucleotides) are detected by differential hybridization of the sample DNA. Illumina on the other hand uses a bead-based technology with slightly longer DNA sequences to detect alleles. The Illumina chips are more expensive to make but provide better specificity.

Aside from the technology, another important consideration is the SNPs that each platform has selected for assay. This can be important depending on the specific human population being studied. For example, it is important to use a chip that has more SNPs with better overall genomic coverage for a study of Africans than Europeans. This is because African genomes have had more time to recombine and therefore have less LD between alleles at different SNPs. More SNPs are needed to capture the variation across the African genome.

It is important to note that the technology for measuring genomic variation is changing rapidly. Chip-based genotyping platforms such as those briefly mentioned above will likely be replaced over the next few years with inexpensive new technologies for sequencing the entire genome. These next-generation sequencing methods will provide all the DNA sequence variation in the genome. It is time now to retool for this new onslaught of data.

## 5. Study Design

Regardless of assumptions about the genetic model of a trait, or the technology used to assess genetic variation, no genetic study will have meaningful results without a thoughtful approach to characterize the phenotype of interest. When embarking on a genetic study, the initial focus should be on identifying precisely *what* quantity or trait genetic variation influences.

### 5.1 Case Control versus Quantitative Designs

There are two primary classes of phenotypes: categorical (often binary case/control) or quantitative. From the statistical perspective, quantitative traits are preferred because they improve power to detect a genetic effect, and often have a more interpretable outcome. For some disease traits of interest, quantitative disease risk factors have already been identified. High-density lipoprotein (HDL) and low-density lipoprotein (LDL) cholesterol levels are strong predictors of heart disease, and so genetic studies of heart disease outcomes can be conducted by examining these levels as a quantitative trait. Assays for HDL and LDL levels, being already useful for clinical practice, are precise and ubiquitous measurements that are easy to obtain. Genetic variants that influence these levels have a clear interpretation – for example, a unit change in LDL level per allele or by genotype class. With an easily measurable ubiquitous quantitative trait, GWAS of blood lipids have been conducted in numerous cohort studies. Their results were also easily combined to conduct an extremely well-powered massive meta-analysis, which revealed 95 loci associated to lipid traits in more than 100,000 people [21]. Here, HDL and LDL may be the primary traits of interest or can be considered intermediate quantitative traits or endophenotypes for cardiovascular disease.

Other disease traits do not have well-established quantitative measures. In these circumstances, individuals are usually classified as either affected or unaffected – a binary categorical variable. Consider the vast difference in measurement error associated with classifying individuals as either “case” or “control” versus precisely measuring a quantitative trait. For example, multiple sclerosis is a complex clinical phenotype that is often diagnosed over a long period of time by ruling out other possible conditions. However, despite the “loose” classification of case and control, GWAS of multiple sclerosis have been enormously successful, implicating more than 10 new genes for the disorder [22]. So while quantitative outcomes are preferred, they are not required for a successful study.

### 5.2 Standardized Phenotype Criteria

A major component of the success with multiple sclerosis and other well-conducted case/control studies is the definition of rigorous phenotype criteria, usually presented as rule list based on clinical variables. Multiple sclerosis studies often use the McDonald criteria for establishing case/control status and defining clinical subtypes [23]. Standardized methods like the McDonald criteria establish a concise, evidence-based approach that can be uniformly applied by multiple diagnosing clinicians to ensure that consistent phenotype definitions are used for a genetic study.

Standardized phenotype rules are particularly critical for multi-center studies to prevent introducing a site-based effect into the study. And even when established phenotype criteria are used, there may be variability among clinicians in how those criteria are used to assign case/control status. Furthermore, some quantitative traits are susceptible to bias in measurement. For example, with cataract severity lens photographs are used to assign cases to one of three types of lens opacity. In situations where there may be disagreement among clinicians, a subset of study records is often examined by clinicians at multiple centers to assess interrater agreement as a measure of phenotyping consistency [24]. High interrater agreement means that phenotype rules are being consistently applied across multiple sites, whereas low agreement suggests that criteria are not uniformly interpreted or applied, and may indicate a need to establish more narrow phenotype criteria.

### 5.3 Phenotype Extraction from Electronic Medical Records

The last few years of genetic research has seen the growth of large clinical bio-repositories that are linked to electronic medical records (EMRs) [25]. The development of these resources will certainly advance the state of human genetics research and foster integration of genetic information into clinical practice. From a study design perspective, identifying phenotypes from EMRs can be challenging. Electronic medical records were established for clinical care and administrative purposes – not for research. As such, idiosyncrasies arise due to billing practices and other logistical reasons, and great care must be taken not to introduce biases into a genetic study.

The established methodology for conducting “electronic phenotyping” is to devise an initial selection algorithm (using structured EMR fields, such as billing codes, or text mining procedures on unstructured text), which identifies a record subset from the bio-repository. In cases where free text is parsed, natural language processing (NLP) is used in conjunction with a controlled vocabulary such as the Unified Medical Language System (UMLS) to relate text to more structured and uniform medical concepts. In some instances, billing codes alone may be sufficient to accurately identify individuals with a particular phenotype, but often combinations of billing and procedure codes, along with free text are necessary. Because every medical center has its own set of policies, care providers, and health insurance providers, some algorithms developed in one clinical setting may not work as well in another.

Once a manageable subset of records is obtained by an algorithm, the accuracy of the results is examined by clinicians or other phenotype experts as gold-standard for comparison. The positive predictive value (PPV) of the initial algorithm is assessed, and based on feedback from case reviewers, the selection algorithm is refined. This process of case-review followed by algorithmic refinement is continued until the desired PPV is reached.

This approach has been validated by replicating established genotype-phenotype relationships using EMR-derived phenotypes [16], and has been applied to multiple clinical and pharmacogenomic conditions [26]–[28].

## 6. Association Test

### 6.1 Single Locus Analysis

When a well-defined phenotype has been selected for a study population, and genotypes are collected using sound techniques, the statistical analysis of genetic data can begin. The *de facto* analysis of genome-wide association data is a series of single-locus statistic tests, examining each SNP independently for association to the phenotype. The statistical test conducted depends on a variety of factors, but first and foremost, statistical tests are different for quantitative traits versus case/control studies.

Quantitative traits are generally analyzed using *generalized linear model* (GLM) approaches, most commonly the Analysis of Variance (ANOVA), which is similar to linear regression with a categorical predictor variable, in this case genotype classes. The null hypothesis of an ANOVA using a single SNP is that there is no difference between the trait means of any genotype group. The assumptions of GLM and ANOVA are 1) the trait is normally distributed; 2) the trait variance within each group is the same (the groups are homoskedastic); 3) the groups are independent.

Dichotomous case/control traits are generally analyzed using either contingency table methods or *logistic regression*. Contingency table tests examine and measure the deviation from independence that is expected under the null hypothesis that there is no association between the phenotype and genotype classes. The most ubiquitous form of this test is the popular chi-square test (and the related Fisher's exact test).

Logistic regression is an extension of linear regression where the outcome of a linear model is transformed using a logistic function that predicts the probability of having case status given a genotype class. Logistic regression is often the preferred approach because it allows for adjustment for clinical covariates (and other factors), and can provide adjusted odds ratios as a measure of effect size. Logistic regression has been extensively developed, and numerous diagnostic procedures are available to aid interpretation of the model.

For both quantitative and dichotomous trait analysis (regardless of the analysis method), there are a variety of ways that genotype data can be encoded or shaped for association tests. The choice of data encoding can have implications for the statistical power of a test, as the degrees of freedom for the test may change depending on the number of genotype-based groups that are formed. *Allelic* association tests examine the association between one allele of the SNP and the phenotype. *Genotypic* association tests examine the association between genotypes (or genotype classes) and the phenotype. The genotypes for a SNP can also be grouped into genotype classes or models, such as dominant, recessive, multiplicative, or additive models [29].

Each model makes different assumptions about the genetic effect in the data – assuming two alleles for a SNP, 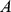 and 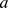, a dominant model (for 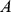) assumes that having one or more copies of the 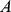 allele increases risk compared to 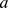 (i.e. 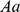 or 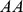 genotypes have higher risk). The recessive model (for 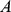) assumes that two copies of the 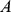 allele are required to alter risk, so individuals with the 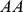 genotype are compared to individuals with 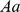 and 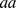 genotypes. The multiplicative model (for 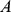) assumes that if there is 3× risk for having a single 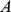 allele, there is a 9× risk for having two copies of the 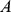 allele: in this case if the risk for 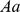 is 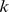, the risk for 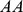 is 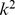. The additive model (for 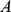) assumes that there is a uniform, linear increase in risk for each copy of the 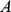 allele, so if the risk is 3× for 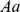, there is a 6× risk for 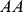 - in this case the risk for 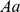 is 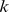 and the risk for 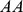 is 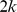. A common practice for GWAS is to examine additive models only, as the additive model has reasonable power to detect both additive and dominant effects, but it is important to note that an additive model may be underpowered to detect some recessive effects [30]. Rather than choosing one model *a priori*, some studies evaluate multiple genetic models coupled with an appropriate correction for multiple testing.

### 6.2 Covariate Adjustment and Population Stratification

In addition to selecting an encoding scheme, statistical tests should be adjusted for factors that are known to influence the trait, such as sex, age, study site, and known clinical covariates. Covariate adjustment reduces spurious associations due to sampling artifacts or biases in study design, but adjustment comes at the price of using additional degrees of freedom which may impact statistical power. One of the more important covariates to consider in genetic analysis is a measure of population substructure. There are often known differences in phenotype prevalence due to ethnicity, and allele frequencies are highly variable across human subpopulations, meaning that in a sample with multiple ethnicities, ethnic-specific SNPs will likely be associated to the trait due to *population stratification*.

To prevent population stratification, the ancestry of each sample in the dataset is measured using STRUCTURE [31] or EIGENSTRAT [32] methods that compare genome-wide allele frequencies to those of HapMap ethnic groups. The results of these analyses can be used to either exclude samples with similarity to a non-target population, or they can be used as a covariate in association analysis. EIGENSTRAT is commonly used in this circumstance, where *principle component analysis* is used to generate principle component values that could be described as an “ethnicity score”. When used as covariates, these scores adjust for minute ancestry effects in the data.

### 6.3 Corrections for Multiple Testing

A p-value, which is the probability of seeing a test statistic equal to or greater than the observed test statistic if the null hypothesis is true, is generated for each statistical test. This effectively means that lower p-values indicate that if there is no association, the chance of seeing this result is extremely small.

Statistical tests are generally called significant and the null hypothesis is rejected if the p-value falls below a predefined alpha value, which is nearly always set to 0.05. This means that 5% of the time, the null hypothesis is rejected when in fact it is true and we detect a *false positive*. This probability is relative to a single statistical test; in the case of GWAS, hundreds of thousands to millions of tests are conducted, each one with its own false positive probability. The cumulative likelihood of finding one or more false positives over the entire GWAS analysis is therefore much higher. For a somewhat morbid analogy, consider the probability of having a car accident. If you drive your car today, the probability of having an accident is fairly low. However if you drive every day for the next five years, the probability of you having one or more accidents over that time is much higher than the probability of having one today.

One of the simplest approaches to correct for multiple testing is the Bonferroni correction. The Bonferroni correction adjusts the alpha value from α = 0.05 to α = (0.05/k) where k is the number of statistical tests conducted. For a typical GWAS using 500,000 SNPs, statistical significance of a SNP association would be set at 1e-7. This correction is the most conservative, as it assumes that each association test of the 500,000 is independent of all other tests – an assumption that is generally untrue due to linkage disequilibrium among GWAS markers.

An alternative to adjusting the false positive rate (alpha) is to determine the false discovery rate (FDR). The false discovery rate is an estimate of the proportion of significant results (usually at alpha = 0.05) that are false positives. Under the null hypothesis that there are no true associations in a GWAS dataset, p-values for association tests would follow a uniform distribution (evenly distributed from 0 to 1). Originally developed by Benjamini and Hochberg, FDR procedures essentially *correct* for this number of expected false discoveries, providing an estimate of the number of true results among those called significant [33]. These techniques have been widely applied to GWAS and extended in a variety of ways [34].

Permutation testing is another approach for establishing significance in GWAS. While somewhat computationally intensive, permutation testing is a straightforward way to generate the empirical distribution of test statistics for a given dataset when the null hypothesis is true. This is achieved by randomly reassigning the phenotypes of each individual to another individual in the dataset, effectively breaking the genotype-phenotype relationship of the dataset. Each random reassignment of the data represents one possible sampling of individuals under the null hypothesis, and this process is repeated a predefined number of times N to generate an empirical distribution with resolution N, so a permutation procedure with an N of 1000 gives an empirical p-value within 1/1000^th^ of a decimal place. Several software packages have been developed to perform permutation testing for GWAS studies, including the popular PLINK software [35], PRESTO [36], and PERMORY [37].

Another commonly used approach is to rely on the concept of *genome-wide significance*. Based on the distribution of LD in the genome for a specific population, there are an “effective” number of independent genomic regions, and thus an effective number of statistical tests that should be corrected for. For European-descent populations, this threshold has been estimated at 7.2e-8 [38]. This reasonable approach should be used with caution, however, as the only scenario where this correction is appropriate is when hypotheses are tested on the genome scale. Candidate gene studies or replication studies with a focused hypothesis do not require correction to this level, as the number of effective, independent statistical tests is much, much lower than what is assumed for genome-wide significance.

### 6.4 Multi-Locus Analysis

In addition to single-locus analyses, genome-wide association studies provide an enormous opportunity to examine interactions among genetic variants throughout the genome. *Multi-locus analysis*, however, is not nearly as straightforward as conducting single-locus tests, and presents numerous computational, statistical, and logistical challenges [39].

Because most GWAS genotype between 500,000 and one million SNPs, examining all pair-wise combinations of SNPs is a computationally intractable approach, even for highly efficient algorithms. One approach to this issue is to reduce or filter the set of genotyped SNPs, eliminating redundant information. A simple and common way to filter SNPs is to select a set of results from a single-SNP analysis based on an arbitrary significance threshold and exhaustively evaluate interactions in that subset. This can be perilous, however, as selecting SNPs to analyze based on main effects will prevent certain multi-locus models from being detected – so called “purely epistatic” models with statistically undetectable marginal effects. With these models, a large component of the heritability is concentrated in the interaction rather than in the main effects. In other words, a specific combination of markers (and only the combination of markers) incurs a significant change in disease risk. The benefits of this analysis are that it performs an unbiased analysis for interactions within the selected set of SNPs. It is also far more computationally and statistically tractable than analyzing all possible combinations of markers.

Another strategy is to restrict examination of SNP combinations to those that fall within an established biological context, such as a biochemical pathway or a protein family. As these techniques rely on electronic repositories of structured biomedical knowledge, they generally couple a bioinformatics engine that generates SNP-SNP combinations with a statistical method that evaluates combinations in the GWAS dataset. For example, the Biofilter approach uses a variety of public data sources with logistic regression and multifactor dimensionality reduction methods [40], [41]. Similarly, INTERSNP uses logistic regression, log-linear, and contingency table approaches to assess SNP-SNP interaction models [42].

## 7. Replication and Meta-Analysis

### 7.1 Statistical Replication

The gold standard for validation of any genetic study is replication in an additional independent sample. That said, there are a variety of criteria involved in defining “replication” of a GWAS result. This was the subject of an NHGRI working group, which outlined several criteria for establishing a positive replication [43]. These criteria are discussed in the following paragraphs.

Replication studies should have sufficient sample size to detect the effect of the susceptibility allele. Often, the effects identified in an initial GWAS suffer from winner's curse, where the detected effect is likely stronger in the GWAS sample than in the general population [44]. This means that replication samples should ideally be larger to account for the over-estimation of effect size. With replication, it is important for the study to be well-powered to identify spuriously associated SNPs where the null hypothesis is most likely true – in other words, to confidently call the initial GWAS result a false-positive.

Replication studies should be conducted in an independent dataset drawn from the same population as the GWAS, in an attempt to confirm the effect in the GWAS target population. Once an effect is confirmed in the target population, other populations may be sampled to determine if the SNP has an ethnic-specific effect. Replication of a significant result in an additional population is sometimes referred to as *generalization*, meaning the genetic effect is of general relevance to multiple human populations.

Identical phenotype criteria should be used in both GWAS and replication studies. Replication of a GWAS result should be thought of as the replication of a specific statistical model – a given SNP predicts a specific phenotype effect. Using even slightly different phenotype definitions between GWAS and replication studies can cloud the interpretation of the final result.

A similar effect should be seen in the replication set from the same SNP, or a SNP in high LD with the GWAS-identified SNP. Because GWAS typically use SNPs that are markers that were chosen based on LD patterns, it is difficult to say what SNP within the larger genomic region is mechanistically influencing disease risk. With this in mind, the unit of replication for a GWAS should be *the genomic region*, and all SNPs in high LD are potential replication candidates. However, continuity of effect should be demonstrated across both studies, with the magnitude and direction of effect being similar for the genomic region in both datasets. If SNPs in high LD are used to demonstrate the effect in replication, the direction of effect must be determined using a reference panel to determine two-SNP haplotype frequencies. For example, if allele 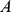 is associated in the GWAS with an odds ratio of 1.5, and allele 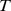 of a nearby SNP is associated in the replication set with an odds ratio of 1.46, it must be demonstrated that allele 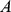 and allele 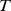 carry effects in the same direction. The most straightforward way to assess this is to examine a reference panel, such as the HapMap data, for a relevant population. If this panel shows that allele 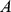 from SNP 1 and allele 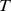 from SNP 2 form a two-marker haplotype in 90% of the sample, then this is a reasonable assumption. If however the panel shows that allele 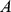 from SNP 1 and allele 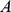 from SNP 2 form the predominant two-marker haplotype, the effect has probably flipped in the replication set. Mapping the effect through the haplotype would be equivalent to observing an odds ratio of 1.5 in the GWAS and 0.685 in the replication set.

In brief, the general strategy for a replication study is to repeat the ascertainment and design of the GWAS as closely as possible, but examine only specific genetic effects found significant in the GWAS. Effects that are consistent across the two studies can be labeled *replicated effects*.

### 7.2 Meta-Analysis of Multiple Analysis Results

The results of multiple GWAS studies can be pooled together to perform a meta-analysis. Meta-analysis techniques were originally developed to examine and refine significance and effect size estimates from multiple studies *examining the same hypothesis* in the published literature. With the development of large academic consortia, meta-analysis approaches allow the synthesis of results from multiple studies without requiring the transfer of protected genotype or clinical information to parties who were not part of the original study approval – only statistical results from a study need be transferred. For example, a recent publication examining lipid profiles was based on a meta-analysis of 46 studies [21]. A study of this magnitude would be logistically difficult (if not impossible) without meta-analysis. Several software packages are available to facilitate meta-analysis, including STATA products and METAL [45], [46].

A fundamental principle in meta-analysis is that all studies included examined the same hypothesis. As such, the general design of each included study should be similar, and the study-level SNP analysis should follow near-identical procedures across all studies (see Zeggini and Ioannidis [47] for an excellent review). Quality control procedures that determine which SNPs are included from each site should be standardized, along with any covariate adjustments, and the measurement of clinical covariates and phenotypes should be consistent across multiple sites. The sample sets across all studies should be independent – an assumption that should always be examined as investigators often contribute the same samples to multiple studies. Also, an extremely important and somewhat bothersome logistical matter is ensuring that all studies report results relative to a common genomic build and reference allele. If one study reports its results relative to allele 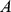 and another relative to allele 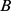, the meta-analysis result for this SNP may be non-significant because the effects of the two studies nullify each other.

With all of these factors to consider, it is rare to find multiple studies that match perfectly on all criteria. Therefore, study heterogeneity is often statistically quantified in a meta-analysis to determine the degree to which studies differ. The most popular measures of study heterogeneity are the 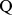 statistic and the 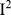 index [48], with the 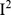 index favored in more recent studies. Coefficients resulting from a meta-analysis have variability (or error) associated with them, and the 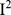 index represents the approximate proportion of this variability that can be attributed to heterogeneity between studies [49]. 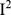 values fall into low (<25), medium (>25 and <75), and high (>75) heterogeneity, and have been proposed as a way to identify studies that should perhaps be removed from a meta-analysis. It is important to note that these statistics should be used as a guide to identifying studies that perhaps examine a different underlying hypothesis than others in the meta-analysis, much like outlier analysis is used to identify unduly influential points. Just as with outliers, however, a study should only be excluded if there is an obvious reason to do so based on the parameters of the study – not simply because a statistic indicates that this study increases heterogeneity. Otherwise, agnostic statistical procedures designed to reduce meta-analysis heterogeneity will increase false discoveries.

### 7.3 Data Imputation

To conduct a meta-analysis properly, the effect of the *same allele* across multiple distinct studies must be assessed. This can prove difficult if different studies use different genotyping platforms (which use different SNP marker sets). As this is often the case, GWAS datasets can be *imputed* to generate results for a common set of SNPs across all studies. Genotype imputation exploits known LD patterns and haplotype frequencies from the HapMap or 1000 Genomes project to estimate genotypes for SNPs not directly genotyped in the study [50].

The concept is similar in principle to *haplotype phasing* algorithms, where the contiguous set of alleles lying on a specific chromosome is estimated. Genotype imputation methods extend this idea to human populations. First, a collection of shared haplotypes within the study sample is computed to estimate haplotype frequencies among the genotyped SNPs. Phased haplotypes from the study sample are compared to reference haplotypes from a panel of much more dense SNPs, such as the HapMap data. The matched reference haplotypes contain genotypes for surrounding markers that were not genotyped in the study sample. Because the study sample haplotypes may match multiple reference haplotypes, surrounding genotypes may be given a score or probability of a match based on the haplotype overlap. For example, rather than assign an imputed SNP a single allele 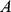, the probability of possible alleles is reported (0.85 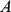, 0.12 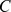, 0.03 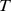) based on haplotype frequencies. This information can be used in the analysis of imputed data to take into account uncertainty in the genotype estimation process, typically using Bayesian analysis approaches [51]. Popular algorithms for genotype imputation include BimBam [52], IMPUTE [53], MaCH [54], and Beagle [55].

Much like conducting a meta-analysis, genotype imputation must be conducted with great care. The reference panel (i.e. the 1000 Genomes data or the HapMap project) must contain haplotypes drawn from the same population as the study sample in order to facilitate a proper haplotype match. If a study was conducted using individuals of Asian descent, but only European descent populations are represented in the reference panel, the genotype imputation quality will be poor as there is a lower probability of a haplotype match. Also, the reference allele for each SNP must be identical in both the study sample and the reference panel. Finally, the analysis of imputed genotypes should account for the uncertainty in genotype state generated by the imputation process.

## 8. The Future

Genome-wide association studies have had a huge impact on the field of human genetics. They have identified new genetic risk factors for many common human diseases and have forced the genetics community to think on a genome-wide scale. On the horizon is whole-genome sequencing. Within the next few years we will see the arrival of cheap sequencing technology that will replace one million SNPs with the entire genomic sequence of three billion nucleotides. Challenges associated with data storage and manipulation, quality control and data analysis will be manifold more complex, thus challenging computer science and bioinformatics infrastructure and expertise. Merging sequencing data with that from other high-throughput technology for measuring the transcriptome, the proteome, the environment and phenotypes such as the massive amounts of data that come from neuroimaging will only serve to complicate our goal to understand the genotype-phenotype relationship for the purpose of improving healthcare. Integrating these many levels of complex biomedical data along with their coupling with experimental systems is the future of human genetics.

## 9. Exercises

1. True or False: Common diseases, such as type II diabetes and lung cancer, are likely caused by mutations to a single gene. Explain your answer.
2. Will the genotyping platforms designed for GWAS of European Descent populations be of equal utility in African Descent populations? Why or why not?
3. When conducting a genetic study, what additional factors should be measured and adjusted for in the statistical analysis?
4. True or False: SNPs that are associated to disease using GWAS design should be immediately considered for molecular studies. Explain your answer.

Answers to the Exercises can be found in Text S1.

**Further Reading**

- 1000 Genomes Project Consortium, Altshuler D, Durbin RM, Abecasis GR, Bentley DR, et al. (2010) A map of human genome variation from population-scale sequencing. Nature 467: 1061–1073.
- Haines JL, Pericak-Vance MA (2006) Genetic analysis of complex disease. New York: Wiley-Liss. 512 p.
- Hartl DL, Clark, AG (2006) Principles of population genetics. Sunderland (Massachusetts): Sinauer Associates, Inc. 545 p.
- NCI-NHGRI Working Group on Replication in Association Studies, Chanock SJ, Manolio T, Boehnke M, Boerwinkle E, et al. (2007) Replicating genotype-phenotype associations. Nature 447: 655–660.

**Glossary**

*GWAS*: genome-wide association study; a genetic study design that attempts to identify commonly occurring genetic variants that contribute to disease risk
*Personalized Medicine*: the science of providing health care informed by individual characteristics, such as genetic variation
*SNP*: single nucleotide polymorphism; a single base-pair change in the DNA sequence
*Linkage Analysis*: the attempt to statistically relate transmission of an allele within families to inheritance of a disease
*Common disease/Common variant hypothesis*: The hypothesis that commonly occurring diseases in a population are caused in part by genetic variation that is common to that population
*Linkage disequilibrium*: the degree to which an allele of one SNP is observed with an allele of another SNP within a population
*Direct association*: the statistical association of a functional or influential allele with a disease
*Indirect association*: the statistical association of an allele to disease that is in strong linkage disequilibrium with the allele that is functional or influential for disease
*Population stratification*: the false association of an allele to disease due to both differences in population frequency of the allele and differences in ethnic prevalence or sampling of affected individuals
*False positive*: from statistical hypothesis testing, the rejection of a null hypothesis when the null hypothesis is true
*Genome-wide significance*: a false-positive rate threshold established by empirical estimation of the independent genomic regions present in a population
*Replication*: the observation of a statistical association in a second, independent dataset (often the same population as the first association)
*Generalization*: the replication of a statistical association in a second population
*Imputation*: the estimation of unknown alleles based on the observation of nearby alleles in high linkage disequilibrium

## Supporting Information

Text S1 Answers to Exercises
(DOCX)
