# Supplementary material for: Chapter 11: Genome-Wide Association Studies
Source: PLoS Comput Biol. 2012 Dec 27;8(12):e1002822. doi: 10.1371/journal.pcbi.1002822 (PMC3531285; doi:10.1371/journal.pcbi.1002822)
Supplement: Text S1 — Answers to Exercises (DOCX) [file pcbi.1002822.s001.docx]

**Exercises**

1. True or False: Common diseases, such as type II diabetes and lung cancer, are likely caused by mutations to a single gene. Explain your answer.

False. Most common diseases do not show strong evidence of linkage within families, indicating that there are not alleles within a single genomic region that are inherited with the disease.

1. Will the genotyping platforms designed for GWAS of European Descent populations be of equal utility in African Descent populations? Why or why not?

No. GWAS genotyping platforms are designed to exploit patterns of linkage disequilibrium. These patterns are population specific, and there is less extensive linkage disequilibrium in African Descent populations due to the higher number of recombination events in that population.

1. When conducting a genetic study, what additional factors should be measured and adjusted for in the statistical analysis?

Genetic studies should account for known clinical and environmental factors to properly estimate the genetic effect due to a SNP, and should also be adjusted for ancestry-derived principal components to correct for potential population stratification.

1. True or False: SNPs that are associated to disease using GWAS design should be immediately considered for molecular studies. Explain your answer.

False. SNPs from GWAS associations may be due to indirect association, where the associated SNP represents the effect of a nearby influential variant. Additional studies should be conducted post-GWAS to narrow the candidate region to a smaller collection of SNPs before conducting molecular validation.
